# The mechanism of action for hyaluronic acid treatment in the osteoarthritic knee: a systematic review

**DOI:** 10.1186/s12891-015-0775-z

**Published:** 2015-10-26

**Authors:** RD Altman, A. Manjoo, A. Fierlinger, F. Niazi, M. Nicholls

**Affiliations:** Division of Rheumatology and Immunology, David Geffen School of Medicine, University of California at Los Angeles, 1000 Veterans Ave, 90024 Los Angeles, CA USA; Division of Orthopaedics, McMaster University, Hamilton, ON Canada; Ferring Pharmaceuticals Inc., Parsippany, NJ USA; Kentucky Orthopaedic and Hand Surgeons, A division of Ortho Kentucky, Lexington, KY USA

**Keywords:** Osteoarthritis, Hyaluronic acid, Therapy, Intra-articular therapy, Systematic review, Mechanism of action

## Abstract

**Background:**

Knee osteoarthritis (OA) is one of the leading causes of disability within the adult population. Current treatment options for OA of the knee include intra-articular (IA) hyaluronic acid (HA), a molecule found intrinsically within the knee joint that provides viscoelastic properties to the synovial fluid. A variety of mechanisms in which HA is thought to combat knee OA are reported in the current basic literature.

**Methods:**

We conducted a comprehensive literature search to identify currently available primary non-clinical basic science articles focussing on the mechanism of action of IA-HA treatment. Included articles were assessed and categorized based on the mechanism of action described within them. The key findings and conclusions from each included article were obtained and analyzed in aggregate with studies of the same categorical assignment.

**Results:**

Chondroprotection was the most frequent mechanism reported within the included articles, followed by proteoglycan and glycosaminoglycan synthesis, anti-inflammatory, mechanical, subchondral, and analgesic actions. HA-cluster of differentiation 44 (CD44) receptor binding was the most frequently reported biological cause of the mechanisms presented. High molecular weight HA was seen to be superior to lower molecular weight HA products. HA derived through a biological fermentation process is also described as having favorable safety outcomes over avian-derived HA products.

**Conclusions:**

The non-clinical basic science literature provides evidence for numerous mechanisms in which HA acts on joint structures and function. These actions provide support for the purported clinical benefit of IA-HA in OA of the knee. Future research should not only focus on the pain relief provided by IA-HA treatment, but the disease modification properties that this treatment modality possesses as well.

## Background

Although osteoarthritis (OA) of the knee is most often a slowly progressive joint disorder, it is one of the leading causes of disability of the adult population [1]. Knee OA, a disease of the entire joint, is characterized by joint pain, cartilage degeneration, and an increase in disability [2]. The progressive nature of OA leads to decreased knee function, affecting an individual’s ability to perform daily activities [3]. Knee OA also negatively impacts socioeconomic factors, as the associated disability often leads to impaired work performance and early retirement [4].

Since there is no established disease modifying agent for OA, there are many options for the treatment of knee OA. Among the pharmacologic therapies, non-steroidal anti-inflammatory drugs (NSAIDs) and intra-articular (IA) corticosteroid injections are most commonly prescribed [5]. These options have inherent limitations, as NSAIDs have potentially serious adverse events associated with their use [6], and IA corticosteroid injections often provide a relatively short period of effective relief [7]. Although corticosteroid injection generally has a positive safety profile, it has been shown to cause a transient increase in blood glucose, which may be a concern for diabetic patients [8]. IA injection of hyaluronic acid (HA) is another treatment option for knee OA pain. HA is nearly ubiquitous in the body, and is a molecule found intrinsically within the knee joint where it provides viscoelastic properties to synovial fluid [9]. As OA progresses, natural HA concentration and the distribution of HA within the joint shifts towards lower ranges of HA molecular weight, leading to a degradation of the mechanical/viscoelastic properties of the endogenous synovial fluid [2, 10, 11]. Lower ranges of molecular weight distributions have also been shown to be strongly correlated to pain [11]. IA-HA administration has aimed to restore this decline in HA concentration and the average molecular weight distribution within the OA knee [9].

IA-HA has been proposed to have many therapeutic mechanisms of action in the OA knee, including shock absorption, joint lubrication, anti-inflammatory effects, chondroprotection, proteoglycan synthesis, and cartilage matrix alterations [2]. The correlation between these various effects has created a better understanding of how IA-HA treatment could provide therapeutic effects for patients with knee OA [12]. There is also evidence suggesting distinct mechanism of action differences between HA products of varying molecular weight. That is, higher molecular weight (HMW) HA has been reported to provide greater anti-inflammatory and proteoglycan synthesis effects, as well as joint lubrication and viscoelasticity maintenance [9, 13]. There also appears to be evidence of variable safety profiles between HA derived through a biological fermentation process (Bio-HA) and avian-derived HA (AD-HA), as AD-HA has the potential for local IA reactions [14, 15].

We aim to summarize the mechanisms of action for IA-HA treatment of knee OA described in the current literature in order to determine the validity of the above mechanisms of action. We will systematically assess and outline the defined mechanisms in which HA may provide a therapeutic benefit, while analyzing reported distinction between product characteristic-dependent effects of IA-HA treatment.

## Methods

### Literature search

We conducted a comprehensive literature search using the MEDLINE, EMBASE, and PubMed databases (Table 1). The search was conducted on May 4^th^, 2014. The inclusion criteria followed throughout the screening process were as follows: 1) Articles describing the mechanism of HA treatment for OA, 2) Articles focused on OA of the knee, and 3) Primary non-clinical basic science articles focussing on “viscosupplementation”/HA treatment. Articles that were published before 1990 or were not published in English were excluded from the study. If multiple studies outlining similar results were published by the same author, only the most recent study was accepted for inclusion.

**Table 1:** Search strategy

MEDLINE and EMBASE – 1470 articles	PubMed – 1395 articles
1. Hyaluronic acid[title]	1. Hyaluronic acid[title]
2. Hylan[title]	2. Hylan[title]
3. Hyaluronan[title]	3. Hyaluronan[title]
4. Viscosupplementation[title]	4. Viscosupplementation[title]
5. 1 or 2 or 3 or 4	5. 1 or 2 or 3 or 4
6. Osteoarthrit*.mp	6. Osteoarthrit*
7. Arthrit*.mp	7. Arthrit*
8. Joint pain.mp	8. Joint pain
9. 6 or 7 or 8	9. 6 or 7 or 8
10. Effect*.mp	10. Effect*
11. Mechanism*.mp	11. Mechanism*
12. 10 or 11	12. 10 or 11
13. 5 and 9 and 12	13. 5 and 9 and 12

*denotes the use of the truncation function within the database

### Data abstraction

Included articles were assessed and categorized based on the mechanism of action described within them. Single articles were included in multiple categories if they analyzed more than one mechanism of HA action within their study design. The mechanism categories utilized in the data abstraction process were: chondroprotection, alterations in proteoglycan and/or glycosaminoglycan (GAG) synthesis, anti-inflammatory effects, mechanical modifications, alterations in subchondral bone, and analgeisic effects.

### Data analysis

The key findings and conclusions from each included article were obtained and analyzed in aggregate with studies of the same assigned category. The results presented have been derived through interpretation of common and consistent mechanism of action presentations within each aforementioned category.

## Results

### Search strategy

**Fig. 1:**
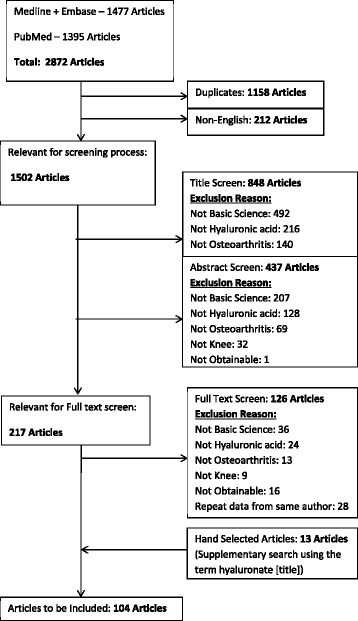
Article screening process

The literature search identified 2,782 potential articles and, of these, 91 articles met the inclusion criteria (Fig. 1). Thirteen additional articles were retrieved by a content expert’s hand search of the literature. This resulted in a total of 104 articles included. The conclusions and key findings of each article were identified within the aforementioned mechanism categories (Table 2).

**Table 2:** Key mechanisms of action reported by the included articles

Mechanism of action	Number of articles	% of included articles	References
Chondroprotective	67	64.42 %	[9, 12, 16–80]
Proteoglycan/Glycosaminoglycan synthesis	22	21.15 %	[27, 42, 44, 48, 62, 69, 83–98]
Anti-inflammatory	21	20.19 %	[13, 20, 21, 25, 27, 52, 54, 55, 68, 72, 96, 97, 99–107]
Mechanical	10	9.61 %	[41, 49, 86, 108–114]
Subchondral bone	8	7.69 %	[23, 31, 32, 45, 115–118]
Analgesic	6	5.76 %	[76, 80, 119–122]

### Chondroprotection

Sixty-seven of the included articles described chondroprotective effects of IA-HA treatment [9, 12, 16–80]. IA-HA has been shown to reduce chondrocyte apoptosis, while increasing chondrocyte proliferation [19, 20]. There are multiple observed effects of IA-HA treatment that produce chondroprotection, many of which are results of HA binding to cluster of differentiation 44 (CD44) receptors. HA binding to CD44 inhibits interleukin (IL)-1β expression, leading to a decline in matrix metalloproteinase (MMP) -1, 2, 3, 9, and 13 productions [32, 34]. This binding to CD44 has been shown to be of greater effect for higher MW HA products [21]. HA also binds to the receptor for hyaluronan mediated motility (RHAMM), which is thought to aid in chondroprotection in addition to CD44 binding [34]. The inhibition of IL-1β expression through CD44 binding is carried out through induction of mitogen-activated protein kinase phosphatase (MKP) -1: a negative regulator of IL-1β [43]. This inhibition of various MMPs impedes catabolic enzyme activity within the joint cartilage [59]. HMW HA was shown to have a greater effect in the inhibitory action of MMP production [21], although these results are unclear, as another study has demonstrated the favourable MMP inhibitory effect of lower molecular weight (LMW) products [72].

Chondrocyte apoptotic events are further decreased by HA-CD44 binding through the reduction of a disintegrin and metalloproteinase with thrombospondin motifs (ADAMTS) expression [38]. These peptidases are involved in the cleavage of important synovial components, including aggrecan, versican, and brevican [81, 82]. Various ADAMTS expression has been shown to decrease as a result of HA-CD44 binding, providing an additional mode of chondroprotection for IA-HA treatment [36, 38, 43]. The production of reactive oxygen species (ROS), such as nitric oxide (NO), results in degeneration of cartilage through increased chondrocyte apoptosis [33]. IA-HA treatment demonstrated a reduction in IL-1β-induced oxidative stress, through inhibition of NO production within the synovium [48, 57]. Additional results of CD44-HA binding resulting in chondroprotective effects mentioned in the current literature include reduction of prostaglandin E2 (PGE2) synthesis [42, 46, 61], and increased heat shock protein 70 (Hsp70) overexpression [25, 70]. These effects similarly provide therapeutic benefit through reduction of chondrocyte apoptosis. It is important to note that HMW HA products demonstrated greater inhibition of PGE2 expression than LMW comparators in a comparative study, resulting in greater chondroprotective effects [61].

### Proteoglycan and glycosaminoglycan synthesis

Twenty-two of the identified studies reported on the enhanced proteoglycan and glycosaminoglycan synthesis related to IA-HA treatment [27, 42, 44, 48, 62, 69, 83–98]. As OA progresses, intrinsic proteoglycan and GAG concentrations decline within the cartilage. Results demonstrated that IA-HA treatment stimulated proteoglycan synthesis, delaying the progression of OA [69, 88]. Aggrecan is the primary proteoglycan within articular cartilage, and IA-HA treatment is shown to both suppress aggrecan degradation, as well as promote intrinsic aggrecan development [62, 93]. IA-HA treatment is shown to mobilize newly synthesized proteoglycan to the outer chondrocyte matrix, potentially providing protection from degradation. Extrinsic HA promoted movement of newly synthesized proteoglycan from the cell-associated matrix to the further-removed matrix in an alginate gel model, which suggests that IA-HA could provide therapeutic relief of OA by strengthening the interterritorial cartilage matrix [92]. A marker of proteoglycan synthesis, sulphate (^35^SO_4_ ), is seen to be increasingly incorporated within chondrocytes after HA introduction [87]. The biological pathway in which HA alters aggrecan levels is shown to be through CD44 and intercellular adhesion molecule (ICAM)-1 binding effects [62]. HMW HA was shown by one study to provide a greater effect of proteoglycan synthesis than LMW HA through stimulation of the insulin-like growth factor (IGF)-1 pathway [89]. IA-HA treatment is also shown to increase endogenous GAG production. IA-HA treatment not only supplemented the synovium with HA, it promoted intrinsic production of HA [27].

### Anti-inflammatory

Twenty-one of the identified studies reported on the anti-inflammatory effects of IA-HA treatment [13, 20, 21, 25, 27, 52, 54, 55, 68, 72, 96, 97, 99–107]. IL-1β is known to demonstrate pro-inflammatory effects, and the aforementioned suppression of IL-1β expression by HA provides anti-inflammatory effects [52]. IL-1β is a key mediator in the anti-inflammatory effects of HA, and is regulated through HA-CD44 binding [59, 104]. IL-1β suppression results in a down-regulation of MMPs as previously mentioned, which also aids in the anti-inflammatory effect of HA [52]. Further suppression of pro-inflammatory mediators IL-8, IL-6, PGE_2_, and tumor necrosis factor (TNFα) provides anti-inflammatory effects of IA-HA treatment [13, 21]. The relation between Toll-Like Receptors (TLR) and an inflammatory response is demonstrated as HA degradation products induced an inflammatory response through CD44 and TLR interaction. This pro-inflammatory response from HA degradation product binding to TLR and CD44 receptors results in increased Nf-kB, IL-1β, TNFα, IL-6, and IL-33 production [105]. HMW HA has been demonstrated to suppress numerous inflammatory mediators through TLR 2 and 4 binding, including TNF-α, IL-1-β, IL-17, MMP-13 and inducible nitrogen oxide synthase (iNOS) [106]. A direct correlation between molecular weight and anti-inflammatory effects has demonstrated larger effects of PGE_2_ and IL-6 inhibition for HMW HA treatment [13]. IL-6 is a pro-inflammatory cytokine, regulated by nuclear factor kappa-light-chain-enhancer of activated B cells (Nf-кB). HA binding to ICAM-1 down regulates Nf-kB, which in turn decreases the production of IL-6 [21, 104]. HMW HA down regulation of TNFα, IL-1B and IL-8 is an additional contributory factor to the anti-inflammatory effects provided by HMW HA [72, 97].

### Mechanical

Ten of the included studies described mechanical effects of HA in the treatment of knee OA [41, 49, 86, 108–114]. The viscous nature of HA treatment is shown to lubricate the joint capsule, preventing degeneration through decreased friction [86]. HA further protects the joint capsule through beneficial shock absorption effects. HA provides cushioning to absorb pressure and vibration within the joint that otherwise may lead to chondrocyte degradation [41]. Osteoarthritic knees are reported to have higher friction within the joint space than healthy knees, which is counteracted by the joint lubrication capabilities that HA possesses [110]. HMW HA has been demonstrated to provide a greater effect of friction reduction due to its viscous properties. The reduction of friction within the joint can provide therapeutic effects, as the cartilage is protected from mechanical degradation [111].

### Subchondral bone

Eight of the included studies outlined the effects that IA-HA has on the subchondral bone [23, 31, 32, 45, 115–118]. It previously has been shown that interaction between subchondral bone osteoblasts and articular cartilage chondrocytes in osteoarthritic joints alters ADAMTS-4/5 and MMP-1, MMP-2, MMP-3, MMP-8, MMP-9, and MMP-13 expression and regulation, mediated by mitogen-activated protein kinase (MAPK) and extracellular signal regulated kinase 1 and 2 (ERK 1/2) signalling pathways [118]. IA-HA also affects the subchondral bone through suppression of MMP-13 and IL-6 via CD44 binding, which potentially prevents abnormal osseous tissue metabolism [116]. The suppression of MMP-13 expression by IA-HA has been suggested to be a critical factor in the effect on OA subchondral bone [117]. The effect that IA-HA has on MMPs, specifically MMP-13, through CD44 binding has been suggested to inhibit the effects of OA within the subchondral bone in numerous non-clinical basic science investigations [32, 45, 116–118]. HA effectively changes subchondral bone density and thickness through trabecular structure alterations, resulting in greater subchondral bone compliancy. This ultimately is shown to reduce the stress put on cartilage during impact loading [23]. An indication of IA-HA stimulation of cartilage/bone interface type II collagen turnover is the increase of urine carboxy-terminal collagen crosslinks (CTX)-II levels observed following IA-HA treatment, which demonstrated improvement in the osteoarthritic knee [31]. Ultimately, HA appeared to limit subchondral bone changes that are characteristic of early OA [115].

### Analgesic

Six of the included articles described the analgesic effects of IA-HA treatment [76, 80, 119–122]. A single injection of HA demonstrated a significant decrease in pain-associated behaviour within a murine model [119]. One study suggested that HA did not directly bind to bradykinin receptors, but provided analgesic effects through interaction with HA receptors and/or free nerve endings within the joint tissue [121]. HA analgesic effects have been shown to occur at mechanosensitive stretch-activated ion channels, where channel activity is significantly decreased upon HA binding [122]. HMW HA was shown to decrease mechanical sensitivity of stretch-activated ion channels, which effectively blocked the pain response. LMW HA was seen to be less effective in blocking this response [122]. HA reduces the action of joint nociceptors, which provides pain reduction within the joint. Sensitized nociceptive terminals within the joint tissue are affected by HA concentration, reducing the pain response exhibited by these terminals [120].

## Discussion

Our review of the existing literature provides a general consensus that IA-HA for knee OA has beneficial effects through several mechanisms of action; however, the predominant mechanism in which therapeutic effect is provided is not clearly understood [2]. In perspective, it is not clear which of these mechanisms are clinically relevant, as it is appreciated that beneficial mechanisms of action are not necessarily transferrable to benefit in the clinical setting. At this time, it is presumed that the clinical benefit of IA-HA in knee OA is due to several concurrent mechanisms of action, instead of any one single specific mechanism of action.

The majority of exogenous HA remains in the joint for a few days; however, the clinical therapeutic effects of HA treatment may be seen for up to 6 months, or more. This may suggest that IA-HA contains disease modifying properties, and does not act solely by restoring viscoelastic properties to the synovial fluid [123]. HA injections may stimulate endogenous production of additional HA by human synoviocytes, aiding in the normalization of HA distribution within the synovial fluid [124]. A large number of reports describe CD44 binding as a primary mode in which HA provides action against knee OA in non-clinical basic science studies. CD44-mediated effects of IA-HA are shown to contribute to the potential chondroprotection, proteoglycan/glycosaminoglycan synthesis, anti-inflammatory, and subchondral mechanisms. This binding is shown to have a variety of effects on numerous signalling pathways, all of which demonstrate some sort of intervention in the progression of OA [13, 21, 32, 36, 38, 42, 43, 46, 59, 61, 104, 116]. The suppression of IL-1β and IL-6 and subsequent effects of this suppression have been suggested to be a key factor in the therapeutic mechanism provided by HA-CD44 binding [123]. It is evident that HA-CD44 binding is a major component in the mechanism in which HA provides therapeutic effect; however, there are additional mechanisms that provide alternate pathways for the effectiveness of HA treatment in OA knees, including ICAM binding, mechanical improvements attributed to shock absorption and lubrication, an increase in cartilage/bone interface type II collagen turnover, as well as analgesic effects through interaction with nerve endings and joint nociceptors [31, 41, 86, 97, 121, 122]. HA binding to the RHAMM receptor promotes wound repair, activates pro-migration and invasion functions, regulates cellular responses to growth factors, and plays a role in fibroblast migration and motility [125–127]. These results of HA-RHAMM binding are potential factors involved in the disease modification effects of HA treatment for OA.

There is evidence which demonstrates that certain intrinsic properties of particular IA-HA products may provide beneficial results in comparison to other IA-HA products. The most recognized of these intrinsic properties is molecular weight. Contrary to a previous basic science review by Ghosh et al., which suggested a potential benefit of LMW HA in providing rheological property restoration over HMW HAs [128], the evidence within the current review has demonstrated advantageous results for HMW HA treatments. The current review supports the view that HMW HA provides superior chondroprotective, proteoglycan and glycosaminoglycan synthesis, anti-inflammatory, mechanical, and analgesic mechanisms of action [61, 89, 97, 111, 122]. A study by Huang et al. demonstrated superior anti-inflammatory effects of HMW HA but superior chondroprotective effects of LMW HA; however, these results regarding chondroprotection are unclear due to lack of additional evidence within the knee OA basic science literature [72]. An increased production of inflammatory cytokines and chemokines, recruitment of inflammatory mediators, and blood vessel formation have been shown to be a response to LMW HA below 500 kDa. While the average MW of available HA products on the market vary greatly, it should be noted that, to our knowledge, all currently available products worldwide have a molecular weight >500 kDa [129, 130]. An analysis of HA-CD44 interaction demonstrated that HA size has direct impact on the affinity in which HA binds to the CD44 receptor [131]. These results demonstrate the capacity of HMW HA to treat the progression of knee OA through CD44 binding of HA. These basic science findings are consistent with systematic reviews of clinical trials and comparative studies which have demonstrated that HMW HA provides greater therapeutic benefit than LMW HA in the treatment of knee OA [6, 132], although the current literature does not provide consensus regarding the clinical efficacy difference between low and high molecular weight HA [133].

Traditionally, HA products had been derived from avian sources; however, some available products are produced by biological fermentation. This process avoids the presence of avian-derived molecules which are suggested to be a potential cause of adverse local reactions [134]. There is a lack of thorough reporting regarding the potential of Bio-HA over AD-HA. One study has suggested AD-HA injection sites may be the cause of synovitis in their patient group, yet the exact pathological agent is unknown [135]. Results from a second study also outline the potential for hylan AD-HA to cause a foreign body giant cell type granulomatous reaction [136]. Research has demonstrated that the flare-ups associated with hylan injection may be correlated to the accumulation of hylan or its breakdown products, as injection site flare ups typically do not occur upon first injection [14]. Avian derived proteins have been shown to be the cause of injection site flare up, as antibodies to chicken serum protein were present in patients who demonstrated injection site adverse reaction after being treated with AD-HA [15]. There is some high-quality clinical evidence that Bio-HA has a significantly smaller incidence of injection site adverse events than AD-HA [134]; however, this is not thoroughly investigated within the current literature. More comprehensive investigation of the difference in incidence of injection site adverse events between Bio-HA and AD-HA from both a basic science and clinical perspective is needed.

This review has methodological strength in its systematic and thorough search of available basic science evidence within multiple databases. The current report also demonstrates rigor in its presentation of multiple mechanisms in which HA acts, providing evidence on all mechanisms whether comprehensively or infrequently reported within the current literature. Limitations of the current study arise due to the subjective classification of included article mechanism of action key conclusions. Included articles may briefly mention alternate mechanisms of action, but were not classified into the corresponding category because the mentioned alternate mechanism was not a key result or conclusion of the study. Future research should analyze the relationship between the various mechanisms presented in this report, and clarify the way in which these mechanisms overlap and may work together to alleviate symptoms of knee OA. Future research should also aim to recognize differences between mechanisms exhibited by high and lower molecular weight products, as well as analyze the safety profile differences between Bio-HA and AD-HA.

## Conclusions

The non-clinical basic science literature provides evidence for numerous mechanisms in which IA-HA may provide clinical benefit in knee OA. Chondroprotection is the most frequently reported mechanism, with HA-CD44 binding being the most frequently reported source of these effects. IA-HA is also reported to provide proteoglycan and glycosaminoglycan synthesis, anti-inflammatory, mechanical, subchondral, and analgesic effects. There is evidence of favorable results for HMW HA treatments in comparison to LMW HA. Bio HA is also demonstrated to provide an advantageous safety profile over AD-HA, as reports demonstrate the association between injection site flare ups and avian-derived proteins. There are a variety of reported mechanisms in which IA-HA is demonstrated to treat knee OA, as well as numerous product characteristics that impact the results of IA-HA treatment. A thorough understanding of the variety of mechanisms in which IA-HA provides beneficial effects within the OA knee, as well as the characteristic-specific effects of various IA-HA products, is required to recognize the applicability and appropriateness of IA-HA treatment for knee OA. Future research should not only focus on the pain relief provided by IA-HA treatment, but the disease modification properties that this treatment modality may possess as well.

## Abbreviations

ADAMTS: A disintegrin and metalloproteinase with thrombospondin motifs
AD-HA: Avian-derived hyaluronic acid
Bio-HA: Biologically-derived hyaluronic acid
CD: Cluster of differentiation
CTX: Carboxy-terminal collagen crosslinks
ERK 1/2: Extracellular signal regulated kinase 1 and 2
GAG: Glycosaminoglycan
HA: Hyaluronic acid
HMW: Higher molecular weight
Hsp70: Heat shock protein 70
IA: Intra-articular
ICAM: Intercellular adhesion molecule
IGF: Insulin-like growth factor
IL: Interleukin
iNOS: Inducible nitrogen oxideLMW, lower molecular weight
MAPK: Mitogen-activated protein kinase
MKP: Protein kinase phosphate
MMP: Metalloproteinase
Nf-кB: Nuclear factor kappa-light-chain-enhancer of activated B cells
NO: Nitric oxide
NSAIDs: Nonsteroidal anti-inflammatory drugs
OA: Osteoarthritis
PGE2: Prostaglandin E2
RHAMM: Receptor for hyaluronan mediated motility
ROS: Reactive oxygen species
TLR: Toll-like receptor
